# Low omega-6 vs. low omega-6 plus high omega-3 dietary intervention for Chronic Daily Headache: Protocol for a randomized clinical trial

**DOI:** 10.1186/1745-6215-12-97

**Published:** 2011-04-15

**Authors:** Christopher E Ramsden, J Douglas Mann, Keturah R Faurot, Chanee Lynch, Syed Taha Imam, Beth A MacIntosh, Joseph R Hibbeln, James Loewke, Sunyata Smith, Rebecca Coble, Chirayath Suchindran, Susan A Gaylord

**Affiliations:** 1 Section on Nutritional Neurosciences, Laboratory of Membrane Biochemistry and Biophysics, NIAAA, NIH, Bethesda, MD, USA; 2 Department of Neurology, School of Medicine, University of North Carolina at Chapel Hill, Chapel Hill, NC, USA; 3 Program on Integrative Medicine, Department of Physical Medicine and Rehabilitation, School of Medicine, University of North Carolina at Chapel Hill, Chapel Hill, NC, USA; 4 North Carolina Translational and Clinical Sciences Institute, University of North Carolina at Chapel Hill, Chapel Hill, NC, USA; 5 Department of Biostatistics, School of Public Health, University of North Carolina at Chapel Hill, Chapel Hill, NC, USA

## Abstract

**Background:**

Targeted analgesic dietary interventions are a promising strategy for alleviating pain and improving quality of life in patients with persistent pain syndromes, such as chronic daily headache (CDH). High intakes of the omega-6 (n-6) polyunsaturated fatty acids (PUFAs), linoleic acid (LA) and arachidonic acid (AA) may promote physical pain by increasing the abundance, and subsequent metabolism, of LA and AA in immune and nervous system tissues. Here we describe methodology for an ongoing randomized clinical trial comparing the metabolic and clinical effects of a low n-6, average n-3 PUFA diet, to the effects of a low n-6 plus high n-3 PUFA diet, in patients with CDH. Our primary aim is to determine if: A) both diets reduce n-6 PUFAs in plasma and erythrocyte lipid pools, compared to baseline; and B) the low n-6 plus high n-3 diet produces a greater decline in n-6 PUFAs, compared to the low n-6 diet alone. Secondary clinical outcomes include headache-specific quality-of-life, and headache frequency and intensity.

**Methods:**

Adults meeting the International Classification of Headache Disorders criteria for CDH are included. After a 6-week baseline phase, participants are randomized to a low n-6 diet, or a low n-6 plus high n-3 diet, for 12 weeks. Foods meeting nutrient intake targets are provided for 2 meals and 2 snacks per day. A research dietitian provides intensive dietary counseling at 2-week intervals. Web-based intervention materials complement dietitian advice. Blood and clinical outcome data are collected every 4 weeks.

**Results:**

Subject recruitment and retention has been excellent; 35 of 40 randomized participants completed the 12-week intervention. Preliminary blinded analysis of composite data from the first 20 participants found significant reductions in erythrocyte n-6 LA, AA and %n-6 in HUFA, and increases in n-3 EPA, DHA and the omega-3 index, indicating adherence.

**Trial Registration:**

ClinicalTrials.gov (NCT01157208)

## Background

Targeted analgesic dietary interventions are a novel strategic approach with promise for alleviating pain and improving quality of life in patients with persistent pain syndromes. Chronic Daily Headache (CDH), defined as presence of headaches lasting 4 hours or more for 15 or more days per month over at least 3 months, is a debilitating pain syndrome that affects 4 to 5% of the adult population in the US, roughly 10 million Americans [1-6]. Loss of work and medical expenses add up to billions of dollars per year [7,8]. Chronic episodic migraines, chronic tension-type headaches, and other primary headaches may evolve into CDH, which is believed to represent a final common pathway for several distinct headache types [9,10]. CDH accounts for up to 40% of patients presenting to headache specialty clinics. Patients with CDH often receive only limited benefit from conventional medical management. Conventional treatments rely heavily on medications that may provide limited or transient relief, target symptoms rather than underlying causes of pain, and are associated with significant side effects and costs [11-15]. Paradoxically, headache medications may facilitate the transformation of intermittent headaches to more frequent and disabling chronic daily headaches [16]. Moreover, medication side effects are often significant and include not only medication overuse syndromes, but also weight gain, fatigue, and depression, among many other side effects [12,13].

### The Role of Diet in Chronic Pain

Multiple metabolic pathways that are important in pain processing converge at the level of omega-6 (n-6) arachidonic acid (AA) metabolism. AA can directly potentiate N-methyl-d-aspartate (NMDA) receptor currents [17-19]. AA is also the precursor to a wide array of neuroactive [20-23] and vasoactive [24-27] compounds that are relevant to pain processing. We hypothesized that the overabundance and/or hyperactive metabolism of n-6 AA in nervous and immune system tissues serve as a fundamental metabolic basis for central pain sensitization and human physical pain [28].

Certain dietary patterns may promote physical pain by increasing the amount and/or subsequent metabolism of AA in nervous and immune system tissues [28]. Humans cannot synthesize AA *de novo*. Tissue phospholipid concentrations of AA (PL-AA) are dependent on dietary intake of 1) n-6 AA [29]; 2) a precursor to AA, linoleic acid (LA) [30]; and 3) competing fatty acids, including n-3 eicosapentaenoic acid (EPA) and docosahexaenoic acid (DHA) [31]. Competition continues as n-6 AA and n-3 EPA + DHA act as rival substrates for enzymatic cleavage and conversion into bioactive compounds (e.g. eicosanoids, docosanoids, endocannabinoids, and endovanilloids). Thus, restricted n-6 consumption and/or increased intake of competing fatty acids may diminish the consequences of elevated tissue concentrations of AA and/or the synthesis of AA metabolites. Restricted n-6 consumption and/or increased n-3 EPA + DHA intake may also increase the abundance and subsequent metabolism of EPA and DHA derivatives with analgesic properties (e.g. resolvins, neuroprotectins) [32].

To test this hypothesis, we designed a 12-week, controlled dietary trial in 76 participants with CDH randomized into 1 of 2 arms: 1) usual medical care plus a low n-6 diet with <2.0 percent of energy (en%) as linoleic acid (LA), <60 mg/d of AA, and average US n-3 intake (Table 1); or 2) usual medical care plus a low n-6, high n-3 diet with <2.0 en% as LA, 1.8 en% as alpha-linolenic acid (ALA) and >1.5 g/d of EPA+DHA (Table 1).

**Table 1:** Nutrient intake targets for a study of exploratory analgesic dietary interventions for chronic daily headache, May 2010*

Nutrient	Low omega-6 diet	Low omega-6 + High omega-3 diet
Total protein	18	18
Total carbohydrate	50	50
Total fat	32	32
Trans fatty acids	<0.5	<0.5
Total saturated fat	13	13
Total monounsaturated fatty acids (MUFA)	16	14
Total polyunsaturated fatty acids (PUFA)	2.5	4.5
Linoleic acid, 18:2n-6 (LA)	<2.0	<2.0
Arachidonic acid, 20:4n-6 (AA)	60 mg	150 mg
Alpha-linolenic acid, 18:3n-3 (ALA)	0.6	1.8
Eicosapentaenoic acid, 20:5n-3 (EPA) + Docosahexaenoic acid, 22:6n-3 (DHA)	150 mg	1500-2000 mg

*All values are expressed as % of energy except AA and EPA+DHA (mg per day)

In this paper, we describe the design of the ongoing dietary trial, and report preliminary compliance data, as indicated by reductions in n-6 LA, AA and the percentage of n-6 highly unsaturated fatty acids (HUFA) in total HUFA (%n-6 in HUFA), and increases in EPA, DHA and omega-3 index in erythrocyte membranes. Implementation strategies are described along with methods for maintaining compliance and ensuring optimal data collection.

## Methods

### Study Design

**Figure 1:**
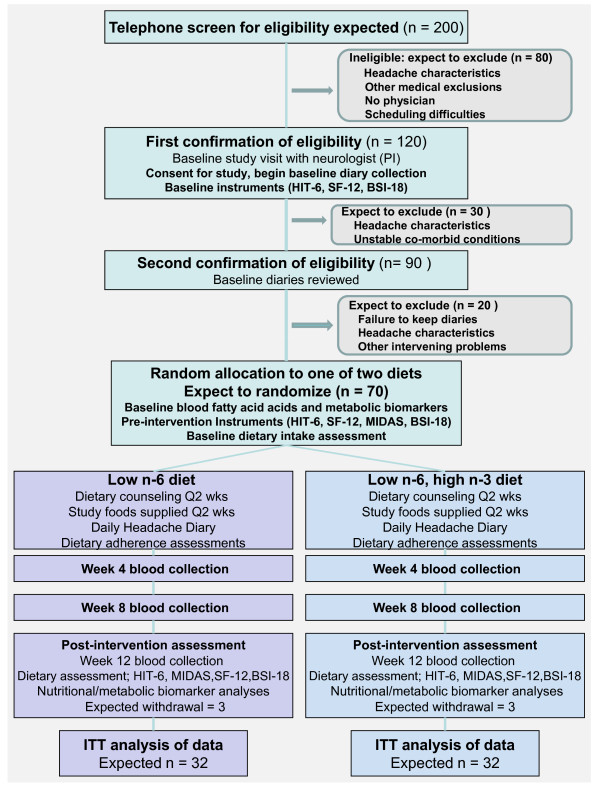
**Study flow diagram for the exploratory analgesic dietary intervention for chronic daily headache, May 2010**. Abbreviations: HUFA, highly-unsaturated fatty acid; HIT-6, Headache Impact Test; MIDAS, Migraine Disability Assessment Questionnaire; BSI-18, Brief Symptom Inventory.

The study is a randomized trial comparing 2 dietary interventions designed to reduce tissue content of AA and total n-6 HUFA, and testing the impact of these interventions on alteration of the phenotypic expression of CDH. Figure 1 illustrates the overall design and subject flow through the study. Study procedures and consent forms were reviewed and approved by the Institutional Review Board and the Clinical and Translational Research Center (CTRC) of the University of North Carolina (UNC).

### Eligibility

Patients referred to the study are screened for CDH using the 2004 International Classification of Headache Disorders (ICHD-II) criteria, and are evaluated for eligibility using the inclusion and exclusion criteria listed in Table 2. Our target is 76 participants qualifying for randomization. Based on our experience with attrition rates in headache studies, we expect at least 80% of the headache sufferers enrolled in the trial to complete the intervention.

**Table 2:** Eligibility of subjects for participation in the study of dietary interventions for chronic daily headache, May 2010

All subjects are evaluated by a neurologist prior to enrollment	
**Inclusion Criteria**	**Exclusion Criteria**

• Subjects >18 years of age	• Marked depression, anxiety or psychosis
• Either gender • Meets 2004 ICHD-II* criteria for Chronic Daily Headache • Headache frequency days/month: > 15 • Headache history: > 2 years Includes: Chronic migraine, transformed episodic migraine into CDH, chronic tension-type headache and persistent daily headache • Willing to complete daily diary for 6 weeks • Able to attend 12 weekly treatments • Under care of physician for headaches • Able to read and communicate in English	• Pregnancy or anticipated pregnancy • Active treatment for a major medical illness, such as malignancy, autoimmune or immune deficiency disorder • History of significant head trauma or head/neck surgery within the past 2 years • History of subarachnoid or intra-cerebral hemorrhage or chronic subdural hematoma • History of clotting disorder • History of systemic infection such as meningitis or encephalitis • History of vasculitis or intracranial mass • Cognitive dysfunction that would prevent informed consent

*International Classification of Headache Disorders, defined by expert members of the International Headache Society

### Recruitment

Participants are recruited from the following sources: 1) University of North Carolina (UNC) Headache Clinic; 2) multiple area neurology practices; 3) broadcast email to UNC employees and students; 4) brochures and flyers at UNC clinics and research centers.

### Screening, consent, and enrollment procedures

Interested individuals contact the research staff for a telephone screening interview. If the screening process identifies them as potentially eligible, the individuals meet with the study staff for consenting, a baseline neurological assessment, and completion of baseline measures. The study neurologist confirms the diagnosis of CDH and excludes from the study those individuals with other medical problems that could put the participant at risk or confound the study results. Individuals may also be excluded if they are unwilling or unable to commit to a 12-week dietary intervention. Excluded patients are referred back to their physicians for further medical management. Enrolled participants continue to be cared for by their personal physicians, but are asked to avoid changes in over-the-counter medications, dietary supplements, and non-pharmacologic treatments for headache during the course of the study. Medication changes for headache management by the treating physician are allowed and documented.

### Baseline Period

Eligible participants are instructed in the maintenance of a daily online headache diary for 6 consecutive weeks while continuing usual care. After they have completed 4 weeks of diaries, their entries are reviewed with the study neurologist to determine eligibility for the treatment phase of the study. Eligibility consists of completion of at least 80% of headache diaries and report of at least 15 headache days per month. Each headache day must have a minimum of 4 consecutive hours of headache. Eligible participants are scheduled to visit the research dietitian for 7 consultations over the course of the 12-week intervention. They are asked to continue their headache diaries for the full 18-week duration of the study.

### Randomization

At the first treatment visit, study participants are randomized to 1 of 2 diets, a low n-6 diet, or a low n-6 plus high n-3 diet (Figure 1 and Table 1), to be maintained for 12 consecutive weeks. Randomization employs a computer algorithm using a random number sequence to generate a permuted block of 4-8 subjects, ensuring equal numbers of participants in each arm of the study [33]. The program, accessed only through an on-line interface by the study dietitian, also documents treatment assignment in an un-editable form with a date stamp.

### Interventions

Participants in both intervention arms receive: 1) targeted and tailored dietitian-administered counseling at the CTRC; 2) access to an intervention-specific website; and 3) a continuous supply of food items with precisely quantified fatty acid compositions formulated to meet nutrient intake targets. Participants meet with the research dietitian approximately every 14 days throughout the 12-week dietary intervention to receive diet education, a 2-week supply of research foods, and a dietary adherence assessment. Meal plans and recipes that meet the nutrient intake targets are provided, as well as specific instructions on how to meet the diet targets and what foods to purchase in addition to those provided by the study. Study-provided foods are selected based on fatty acid analysis completed by the Section on Nutritional Neurochemistry, Laboratory of Membrane Biochemistry and Biophysics, NIAAA/NIH. Research foods are procured and prepared by the Nutrition Research and Metabolism Core of the Clinical and Translational Research Center at UNC, which is supported in part by grant UL1RR025747 from the National Center for Research Resources, National Institutes of Health.

Participants in both diet groups are educated on how to limit LA and AA in their diets. Because most available US oils contain substantial amounts of LA, participants are provided with, and instructed to use, only low LA oils and fat sources (e.g. macadamia nut oil, coconut oil, low-LA olive oil, butter, fat-free mayonnaise, specified salad dressings). Nutrient analysis performed on numerous brands of each of the above oils and fat sources ensured the selection of those with the lowest LA content.

Participants randomized to the low n-6 plus average n-3 diet group are instructed to reduce their intake of AA by restricting consumption of egg yolks, organ meats, meats, and certain fish. They are asked to eat up to 2 vegetarian meals per day, and up to 1 meal per day that includes very lean (low n-3) fish or shellfish and/or egg whites. Study foods provided to low n-6 dieters include several types of canned beans, frozen lean seafood (e.g. shrimp, grouper, cod, very low-fat chunk light tuna), and low-fat turkey slices, chosen on the basis of nutrient analyses to achieve the lowest amounts of n-3 EPA+DHA and n-6 AA.

Study participants randomized to the low n-6 plus high n-3 diet are instructed to consume 14 g of ground flaxseed each day, to reach the nutrient intake target for n-3 ALA (Table 1). They receive ground flaxseed or prepared flaxseed products to include granola, muffins, granola bars and bean dip. To reach EPA and DHA nutrient intake targets, the low n-6 plus high n-3 dieters are counseled to consume 1-2, 4 oz servings of fatty fish per day. They are provided with high n-3 canned tuna, canned salmon, frozen salmon fillets, frozen trout and sardines. Nutrient analysis of numerous seafood sources enabled selection of foods with the highest amounts of n-3 EPA+DHA.

Participants in both diet groups are given breads, crackers, tortillas, popcorn, yogurt, string cheese, frozen fruits and vegetables, beans, and prepared vegetarian chili. These carefully-selected study foods displace high LA foods and facilitate adherence with study diets. Web-based intervention materials complement and reinforce dietitian advice. Diet education materials include: 1) Diet Guidelines; 2) Seven-day Meal Plan; 3) Grocery Shopping Guides; 4) Dining Out Guide; 5) Food Lists; and 6) more than 50 recipes that either utilize the study provided foods or meet study guidelines.

Participants complete self-reported outcome assessments at baseline, at their first intervention visit, and at the end of the intervention. They have blood drawn for biomarkers at the first intervention visit (Week 6), and again at weeks 10, 14, and 18. The research staff validates data through weekly contact with participants.

### Masking

Because of the nature of the interventions, the research dietitian cannot be masked to treatment condition. Similarly, participants are aware of the foods included in their diets. However, participants do not have access to information about the other diet. In addition, steps are taken to ensure that credibility of both dietary interventions is maintained. A questionnaire assessing the credibility of both diets as an intervention for headache is administered after the first visit with the dietitian. All investigators except the research dietitian are masked to treatment assignment. Similarly, participants' personal physicians are masked to treatment assignment.

### Remuneration

Participants receive 12 weeks of dietary treatment at no cost. During these 12 weeks, participants receive food for 2 meals and 2 snacks per day, supplied every other week by the CTRC research kitchen. Participants completing the study receive $145 in compensation in addition to costs of mileage and parking of up to $13 per visit.

### Outcome measures and study instruments

#### Primary Outcomes

The primary outcome (Aim 1) is the change in percentage of n-6 in HUFA in total HUFA (%n-6 in HUFA) in plasma and erythrocyte lipid pools, from pre-intervention (Week 6 - end of baseline phase) to post-intervention (Week 18). For determination of the %n-6 in HUFA, fatty acids that are at least 20 carbons in length with 3 more double bonds are considered to be HUFAs. The equation for calculation of %n-6 in HUFA is 100 × (20:3n-6 + 20:4n-6 + 22:4n-6 + 22:5n-6)/(20:5n-3 + 22:5n-3 + 22:6n-3 + 20:3n-6 + 20:4n-6 + 22:4n-6 + 22:5n-6 + 20:3n-9). For Aim 2, we examine the rate of change in %n-6 in HUFA over the 12 weeks of the intervention as measured at Study Week 6, Week 10, Week 14, and Week 18 (Weeks 0, 4, 8, and 12 of the intervention phase).

#### Secondary Laboratory Outcomes

Secondary laboratory outcomes include individual n-6 and n-3 fatty acids in plasma and erythrocyte lipid pools, including n-6 linoleic acid (18:2n-6) and AA, as well as n-3 EPA and DHA. Lipid mediators derived from n-6 AA, EPA and DHA (e.g. eicosanoids, docosanoids), and mediators that may be indirectly influenced by n-6 and n-3 HUFA status (e.g. cytokines, pro-inflammatory enzymes, substance P), will be measured at study completion.

Changes in inflammatory gene expression in peripheral blood mononuclear cells (PBMCs) will be assessed in an additional pilot study. We will test: (1) the effects of the low n-6 diet compared to baseline dietary conditions (gene expression before the dietary intervention); (2) the effects of the low n-6 + high n-3 diet compared to baseline; and (3) the effects of the low n-6 versus the low n-6 + high n-3 diet compared to baseline. PBMCs are isolated from whole blood and RNA will be extracted utilizing Qiagen RNeasy Micro Kit (Valencia, CA). The RNA yield will be quantified with Nanodrop ND 1000 spectrophotometer (Nanodrop Technologies, Wilmington, DE), and integrity will be measured with an Agilent 2100 Bioanalyzer with RNA 6000 Nano chips (Santa Clara, CA). The expression of 84 key genes involved in the inflammatory response will be assessed using the Human Inflammatory Cytokines & Receptors RT² Profiler PCR Array (SA Biosciences, Frederick, MD).

#### Clinical outcomes

Although this study is not powered to assess changes in clinical endpoints, we are measuring the following outcomes in preparation for a larger, fully powered trial of clinical efficacy (Table 3):

**Table 3:** Summary of measures and timing of administration for exploratory analgesic dietary intervention for chronic daily headache as of May 2010

Variable or Instrument	Usual Care - UC Weeks 0 to 6	UC + Diet Weeks 6 to 18
***Primary outcome***		

Headache QOL - HIT-6 §	Week 0, Week 6	Week 18
***Secondary outcomes***		

Headache Diary: Frequency	Daily	Daily
Perceived clinical change	Week 0, Week 6	Week 18
Headache disability - MIDAS#	Week 6	Week 18
Headache Diary: Intensity/Duration	Daily	Daily
Headache Diary: Sleep Duration	Daily	Daily
Headache Diary: Medication Use/Cost	Daily	Daily
Headache Diary: Health care visits	Daily	Daily
Satisfaction with care	Week 0, Week 6	Week 18
Psychological health: BSI-18	Week 0, Week 6	Week 18
Health status: Rand SF-12	Week 0, Week 6	Week 18
***Process measures***		

Nutritional and Metabolic Biomarkers	Week 6	Weeks 10, 14, and 18
Dietary Intake Assessment	Week 5-6	Week 16-18
Food Intake Diary		3 days every 4 weeks
BMI, BP, Weight	Week 5-6	Weeks 10, 14, and 18
***Effect modification measures***		

Demographics	Week 0	
Clinical Characteristics	Week 0	
Expectation of benefit†		After dietary instruction

*International Classification of Headache Disorders, defined by expert members of the International Headache Society

† Based on a measure developed by Borkovec and Nau to measure credibility of psychological interventions.

§ Headache Impact Test was developed to measure headache-related quality of life.

# Migraine disability assessment score assesses the number of days of missed work or school in the past 3 months.

(1) Headache frequency and intensity, medication use, cost of medication, and health care utilization, all collected by self-report in a detailed daily headache diary [34]. The diary, available to participants on a secure website, collects data on headache intensity (none, mild, moderate, severe) and associated aura for every hour of the day and night.

(2) Headache-specific quality-of-life, measured through the Headache Impact Test (HIT-6) [35] at the time of randomization and after completion of the intervention. The HIT-6, with good internal reliability [36], covers functioning relevant to headache-related disability: pain, social functioning, role functioning, vitality, cognitive functioning, and psychological distress. A decrease of 2.3 HIT points (95% CI, 0.3 to 4.3) over 6 weeks among patients with chronic headache corresponds to a "somewhat better" self-reported rating on a global clinical change scale [36].

(3) Headache-related disability, measured with the Migraine Disability Assessment Questionnaire (MIDAS) [37,38] pre-randomization and at the end of the 12-week intervention. Derived from the Headache Impact Test, MIDAS is a 7-item questionnaire that assesses the number of days during the previous 3 months that respondents missed work or school, experienced decreased productivity at work or home, or missed social engagements because of headaches [38]. Test-retest reliability and internal consistency are acceptable (r_2_: 0.67-0.73; alpha = 0.83) [39].

(4) General health-related quality-of-life (SF-12) is measured with a shorter version of the popular Medical Outcomes Study Short Form Health Survey (SF-36) that yields a summary score for mental components and physical components [40]. The SF-12 was found to be reliable, valid, and responsive to change in a population of chronic-back-pain patients [41], and has been employed in migraine research [42].

(5) General psychological distress as measured with the Brief Symptom Inventory (BSI-18), a short (18-item) version of the Symptom Checklist-90-R (SCL-90-R) [43]. Subjects are asked to rate how much they were bothered in the last 7 days by each of 18 symptoms, including separate scores for anxiety, depression, somatization, and a global symptom severity index. The BSI has good internal consistency and test-retest reliabilities (alpha = 0.71-0.85; r = 0.68 - 0.91)[44].

#### Possible effect modifiers

Effect modifiers consist of demographics and headache-history variables such as: (1) clinical characteristics of headache over time; (2) use of prescribed and over-the-counter medications; (3) associated medical conditions; (4) family history of headache and other disorders; and (5) a single-item assessment of satisfaction with care. Subject credibility and expectations are assessed using an instrument adapted from a validated scale developed by Borkovec and Nau [45,46] and administered just after the first intervention visit. Because high expectations of treatment are closely related to better outcomes [47,48], data analysis will test whether participants in the 2 groups have similar or different expectations of benefit.

#### Process Measures

The research dietitian collects a 3-day food record on visit 3 and daily food-intake checklists every 2 weeks thereafter. Each week, participants rank their diet adherence on a scale of 1 to 5. The research dietitian rates diet adherence on a scale of 1 to 5 based on the study-diet nutrition counseling session every other week. Dietary intake assessments (24-hour recall) are performed on 3 non-consecutive days during the baseline phase and repeated during the final 3 weeks of the intervention phase. Blood samples are obtained for fatty acid analysis at baseline and every 4 weeks during the intervention phase.

### Quality control

All personnel complete lab safety training and step-by-step instruction in lab processing. Research assistants record the times that the sample is drawn and the time of each processing step, including the time to centrifuge and time to freezer (-80 F or liquid N tank) storage (Eppendorf) tubes. Each specimen is bar-coded, and triple-recorded to ensure accurate identification. For metabolites sensitive to time, expedited processing ensures good sample quality.

### Adverse events

Reports of adverse events are obtained from participants at each intervention visit, from self reports recorded in the headache diaries, or by direct contact via email or phone with study staff. Adverse events are investigated by the study neurologist and are reported to the UNC Institutional Review Board.

### Statistical Analysis

#### Overview

This study is a 2-arm intervention trial with a baseline measurement and with repeated measures of the outcome variable over time. The primary outcome variable is %n-6 in HUFA after dietary interventions. To correct for violations of the underlying assumptions of regression (e.g., normality, variance homogeneity), we plan to use a transformed value of the proportions as our dependent variable (angular, logit, or power transformation). We plan to invoke intent-to-treat analysis in dealing with missing data.

#### Analysis of Aim 1

In Aim 1, we will assess the extent of lowering %n-6 in HUFA after 12 weeks of targeted dietary interventions. All participants contribute specimens at randomization (week 6), and at weeks 10, 14 and 18. As a first step, we will test the equality of the transformed mean proportions after 12 weeks of the intervention with a t-test and will use the transformed proportions in a regression model to compare the treatment effects adjusting for covariates, including the baseline proportion and other variables that may influence the accumulation and turnover of LA in adipose tissue, such as age, sex, and baseline BMI.

#### Analysis of Aim 2

To test Aim 2, we will make use of the repeated measures of the outcome variable measured at 6, 10, 14, and 18 weeks to compare the rate of reduction in the %n-6 in HUFA over time in the 2 groups. Because the measurements within each person over time are correlated, we will use a hierarchical linear model with transformed proportions as the outcome variable. The first-level model specifies the outcome as a linear function of time. In the second level, the slope and intercept will be a linear function of the treatment variable. The random effects will represent the within- and between-group variances. The regression coefficients of the time-by-treatment terms are of interest here. If the regression coefficients of the interactions are not statistically significant from zero, we will conclude that the rate of change in the outcome variable over time is the same in both groups.

#### Secondary Laboratory Aims

The study will explore multiple secondary aims. We will examine the metabolic impact of dietary interventions by examining the association between the plasma concentrations of n-6 AA-derived mediators and %n-6 in HUFA. We will employ a mixed model with repeated measurements of mediators as the outcome and with %n-6 in HUFA (transformed), time, and time by %n-6 in HUFA, to formally test the association between the two variables and the time trends.

We plan to analyze the impact of the dietary interventions on inflammatory gene expression. To estimate changes, we will analyze log fold-change as a continuous response. The hypothesis of no change in mean response will be tested at the 0.05/80 level, using a paired t-test, for each of the 84 genes correcting for multiple comparisons.

#### Secondary Clinical Aims

We will also examine clinical data, including the diary data and the HIT-6 score. The 24-hour daily diary, successfully used in a previous UNC study, collects hourly headache experience for 18 weeks [34]. To examine the role of headache frequency, we will use the diary data to construct summary measures of headache experience for each day, including the number of headache hours in a day and the number of hours with moderate-severe headaches. With the repeated measurements of headache hours per day as the outcome variable, we will construct random-effects Poisson regression models with treatment as the exposure, and age and sex as covariates, to examine the association between the diets, blood fatty acids and their derivatives, and headache.

To test the effects on quality-of-life, we will construct a regression equation with the final HIT-6 score as the outcome variable, the treatment group as the exposure variable and the baseline HIT score as the control variable. We will also test a limited number of interaction effects to see whether the treatment effects vary by sub-groups (e.g., gender, age). In addition, controlling for the baseline score, we will examine the relationship between the post-intervention HIT score and post-intervention %n-6 in HUFA.

#### Handling of Missing Data

Every effort will be made to contact participants who fail to complete required study instruments. Based on our experience with UNC headache patients, we anticipate that only a small proportion will not complete the study. Through missing information evaluation, we will assess whether drop-outs depend on any patient characteristics. If necessary we will do multiple imputations in the analysis.

#### Power calculations

Assuming that the %n-6 in HUFA will be on average 0.8, 0.5 and 0.3 at the end of the 12 weeks (as predicted by Lands empirical equation [31] and the nutrient intake targets) and a common standard deviation set at the maximum value of 0.5, we determined that, to achieve 80% power, we need 32 individuals per each arm. Repeated measures power-analysis procedures indicate that we will have adequate power (close to 80%) to test the time interaction effect with 32 persons measured over 4 time points (Aim 2).

## Results

Recruitment into the study has proceeded as planned (Table 4). The majority of participants have been referred by their health care provider, particularly those who see a neurologist. Other participants are self-referred, having learned about the study through UNC advertisements, brochures, flyers, friends and family.

**Table 4:** Recruitment for the exploratory analgesic dietary intervention for chronic daily headache, May 2010

	N	Percent
Inquiries about study	183	
Potentials to be screened	59	32.2
Screened	124	67.8
Eligible, but declined to participate	5	4.0
Ineligible at telephone screen	35	28.2
Headache characteristics	15	42.9
Medical/psychiatric problems	4	11.4
Logistic problems (time/distance)	11	31.4
Dietary Issues	2	5.7
Other/unknown	3	8.6
Scheduled for baseline consent	84	68.3
Ineligible at baseline consent	5	6.0
Eligible at baseline consent	79	94.0
Withdrew/ineligible before randomization	10	11.9
Withdrew after randomization	5	6.0
Awaiting baseline visit	18	14.5
Enrolled	46	37.9
Referral pattern		
Health professionals*	126	72.0
Electronic advertisements	32	18.3
Print advertisements	12	6.9
Family/Friends	2	1.1
Prior Study	3	1.7
Unknown	8	

* Health professionals that referred patients to the study included neurologists, family physicians, and nurses.

At this time, 124 individuals have been screened for eligibility of which 63 were enrolled into the study. Five enrolled individuals were ineligible to begin the dietary intervention due to low headache frequency (3), an intervening illness (1), or failure to keep up with the diaries. In addition to those who did not qualify, 10 individuals withdrew after giving consent, 5 during the baseline phase and 5 during the diet phase. Of the 5 individuals who withdrew during the baseline phase, 4 cited scheduling difficulties and 1 moved out of town. Of the 5 individuals who withdrew after randomization, 3 cited difficulty adjusting to the diet, 1 became pregnant, and another withdrew due to a traumatic injury that was unrelated to the study diet.

Adherence with the daily diary has been excellent, with 83.3% of participants completing at least 80% of daily entries. Most have tolerated the diets well; they have demonstrated diet adherence via changes in erythrocyte fatty acids. Preliminary analysis of composite erythrocyte fatty acid data of the first 20 participants, with blinding preserved (i.e., both diet groups are analyzed together and investigators remain masked to diet group), reveals significant reductions in %n-6 in HUFA and n-6 AA and LA, and significant increases in n-3 EPA, DHA and the omega-3 index compared with baseline erythrocyte fatty acids over the 12-week intervention (Table 5).

**Table 5:** Means of erythrocyte fatty acids over the 12-week intervention, Chronic Daily Headache study, May 2010

	Baseline (n = 20)		Week 4 (n = 20)		Week 8 (n = 20)		Week 12 (n = 18)	
	
Variable	Mean	95% CI	Mean	95% CI	Mean	95% CI	Mean	95% CI
Omega-3 index	4.4	3.9, 5.0	6.2	5.4, 7.1	7.0	5.9, 8.1	7.8	6.5, 9.0
% n-6 in HUFA*	76.3	74.5, 78.0	70.4	67.5, 73.2	67.4	63.7, 71.0	64.7	60.3, 69.0
DHA	4.0	3.5, 4.5	5.2	4.5, 5.8	5.7	4.9, 6.5	6.3	5.4, 7.3
EPA	0.46	0.39, 0.54	1.1	0.8, 1.3	1.3	0.95, 1.6	1.4	1.0, 1.8
AA	14.8	14.5, 15.2	14.4	13.7, 15.1	13.8	13.2, 14.5	13.7	12.9, 14.5
LA	11.5	10.8, 12.1	9.5	8.8, 10.2	9.3	8.6, 10.0	9.5	8.8, 10.3

* %n-6 in HUFA is the percentage of omega-6 fatty acid in total HUFA as calculated by: 100 × (20:3n-6 + 20:4n-6 + 22:4n-6 + 22:5n-6)/(20:5n-3 + 22:5n-3 + 22:6n-3 + 20:3n-6 + 20:4n-6 + 22:4n-6 + 22:5n-6 + 20:3n-9).

Abbreviations: HUFA, highly-unsaturated fatty acid; DHA, Docosahexaenoic acid; EPA, eicosapentaenoic acid; AA, arachidonic acid; LA, linoleic acid.

Based on our current rates of recruitment, we expect to have a sufficient number of participants to complete the study 2 1/2 years after the first patient was enrolled. Based on these numbers, we should have adequate power to test our hypothesis of differential %n-6 HUFA in both plasma and erythrocyte lipid pools. Examination of the clinical data of the study (HIT-6, daily diaries) should allow us to predict effect sizes for a larger clinical trial.

## Discussion

This protocol paper provides a detailed overview of design and implementation of a randomized, outpatient, clinical trial comparing two novel analgesic dietary interventions as a strategy for management of CDH. Results so far indicate excellent adherence with both diets, enabled by provision of food for more than 2 meals per day, expert dietary counseling, and extensive web-based educational tools to support the interventions. Participants in the study have demonstrated a willingness to keep study appointments, adhere to the diets, and complete daily headache diaries. In addition, quality control procedures have ensured that laboratory specimens are meeting the highest standards for processing.

Preliminary blinded analysis of erythrocyte fatty acid data indicates significant reductions in n-6 AA and %n-6 in HUFA, and significant increases in n-3 EPA, DHA and the omega-3 index (Table 5). These changes are statistically significant despite the composite analysis of both dietary groups, albeit with greater variability than expected for one diet alone. Importantly, it is not possible to determine if %n-6 in HUFA and n-6 AA were reduced in both, or only one, diet group from this preliminary analysis. It is also worth noting the significant reduction of n-6 LA in these first 20 participants. To our knowledge, this is the first demonstration of reduced LA in human tissues with dietary LA intake below 2 en%. Recently, metabolic derivatives of LA, including 9- and 13-hydroxyoctadecadienoic acid (9- and 13-HODE), were identified as an endogenous family of potent TRPV1 (endovanilloid) receptor agonists [49,50]. TRPV1 receptors are critically involved in the initiation and perpetuation of chronic pain, and TRPV1 antagonists have potent analgesic effects [51]. Therefore, the long-term consumption of diets that contain less than 2 en% as LA could promote analgesia by reducing the LA content in human tissues and subsequently down-regulating the synthesis of LA-derived TRPV1 agonists. The long-term consumption of the low n-6, and low n-6 plus high n-3 diets in our current trial could have important implications for central pain sensitization and human physical pain via altering the abundance and metabolism of LA, AA, EPA, and/or DHA. Full analyses of metabolic and clinical outcomes at study completion will provide novel data to help answer the following open questions:

1) Does the reduction of dietary n-6 LA and AA over a 12-week period decrease the abundance of LA and AA, and increase n-3 EPA and DHA, in human erythrocytes and plasma?

2) Does the reduction of dietary n-6 LA and AA decrease circulating levels of bioactive mediators derived from LA (e.g. endovanilloids) and AA (e.g. eicosanoids, endocannabinoids)?

3) Does the addition of n-3 ALA, EPA and DHA to low n-6 diets produce additional metabolic alterations?

4) Do low n-6, average n-3 diets and/or low n-6, high n-3 diets attenuate human physical pain? If so, are pain reductions correlated with alterations in tissue contents of specific fatty acids and/or their metabolic derivatives?

### Limitations

The primary methodological limitation for interpreting the primary outcome (%n-6 in HUFA) and other metabolic outcomes of this trial is the limited duration of 12 weeks. Industrialized populations consuming large quantities of LA-rich seed oils maintain large quantities of n-6 LA in adipose tissue. In the US, adipose tissue LA increased from about 6% of total fatty acids in 1960 [52,53] to about 18% in 1986 [54], which coincided with an increase in dietary LA from about 2 en% in 1909 to 7 en% in 1999 [55]. Because n-6 and n-3 fatty acids have a half-life of about 1 to 2 years in adipose tissue [56-58], the 12-week dietary period employed in this trial may not be of sufficient duration for plasma and erythrocyte PUFAs to reach dynamic equilibrium. Our preliminary blinded analysis of erythrocyte fatty acid data is consistent with the expectation that maximal reductions in plasma and tissue LA and/or AA may not occur over 12 weeks; AA and %n-6 in HUFA continued to decline, and EPA, DHA, and the omega-3 index continued to increase between 8 and 12 weeks (Table 5).

The primary methodological limitation for interpreting the clinical outcomes of this trial is the lack of a control group consuming average US amounts of individual n-6 and n-3 fatty acids. This will limit the ability to determine if changes in clinical outcomes (headache frequency, intensity, and quality-of-life) are due to the diets themselves, to the expectation of benefits associated with participation in a clinical trial, or to the placebo effect of the interventions. This study was not designed to test the clinical efficacy of the dietary interventions. However, the study does demonstrate that, with support, free-living subjects can successfully modify their diets enough to result in significant changes in erythrocyte fatty acids, setting the stage for a larger, fully powered randomized controlled trial of clinical efficacy in CDH or another chronic pain population.

## Competing interests

The authors declare that they have no competing interests.

## Authors' contributions

CER, JDM, KRF, BM and SG were involved in developing the original study design and application for funding. JDM is the PI and the primary clinician on the funded proposal, with all other authors as co-applicants on the proposal. BM the lead dietitian on the protocol, developed the study diets in collaboration with CER. CL and  STI helped create the intervention materials.CS is providing oversight of data collection and statistical assessments. JRH provided expertise in fatty acid analysis of foods and blood, study design, and interpretation of results. SS is the lead investigator of the pilot study of inflammatory gene expression. All authors participated in development of research protocols and design of the study. All authors contributed to the writing and approved the final manuscript.
